# Peptic Ulcer Disease: A Brief Review of Conventional Therapy and Herbal Treatment Options

**DOI:** 10.3390/jcm8020179

**Published:** 2019-02-03

**Authors:** Lucija Kuna, Jelena Jakab, Robert Smolic, Nikola Raguz-Lucic, Aleksandar Vcev, Martina Smolic

**Affiliations:** 1 Department of Pharmacology and Biochemistry, Faculty of Dental Medicine and Health Osijek, Josip Juraj Strossmayer University of Osijek, 31000 Osijek, Croatia; lucija.kuna@fdmz.hr (L.K.); nikola.rlucic@gmail.com (N.R.-L.); 2 Department of Pathophysiology and Physiology with Immunology, Faculty of Dental Medicine and Health Osijek, Josip Juraj Strossmayer University of Osijek, 31000 Osijek, Croatia; jelena.jakab@fdmz.hr (J.J.); robert.smolic@fdmz.hr (R.S.); aleksandar.vcev@fdmz.hr (A.V.); 3 Department of Internal Medicine, Faculty of Medicine Osijek, Josip Juraj Strossmayer University of Osijek, 31000 Osijek, Croatia; 4 Department of Pharmacology, Faculty of Medicine Osijek, Josip Juraj Strossmayer University of Osijek, 31000 Osijek, Croatia; 5 Department of Internal Medicine, University Hospital Osijek, 31000 Osijek, Croatia

**Keywords:** peptic ulcer disease, *Helicobacter pylori* infection, herbal treatment

## Abstract

Peptic ulcer is a chronic disease affecting up to 10% of the world’s population. The formation of peptic ulcers depends on the presence of gastric juice pH and the decrease in mucosal defenses. Non-steroidal anti-inflammatory drugs (NSAIDs) and *Helicobacter pylori (H. pylori)* infection are the two major factors disrupting the mucosal resistance to injury. Conventional treatments of peptic ulcers, such as proton pump inhibitors (PPIs) and histamine-2 (H2) receptor antagonists, have demonstrated adverse effects, relapses, and various drug interactions. On the other hand, medicinal plants and their chemical compounds are useful in the prevention and treatment of numerous diseases. Hence, this review presents common medicinal plants that may be used for the treatment or prevention of peptic ulcers.

## 1. Introduction

Peptic ulcer is an acid-induced lesion of the digestive tract that is usually located in the stomach or proximal duodenum, and is characterized by denuded mucosa with the defect extending into the submucosa or muscularis propria [1]. The estimated prevalence of peptic ulcer disease in the general population is 5–10% [2], but recent epidemiological studies have shown a decrease in the incidence, rates of hospital admissions, and mortality associated with peptic ulcer [3,4]. This is most likely secondary to the introduction of new therapies and improved hygiene, which resulted in a decline in *Helicobacter pylori (H. pylori)* infections. 

Traditionally, mucosal disruption in patients with the acid peptic disease is considered to be a result of a hypersecretory acidic environment together with dietary factors or stress. Risk factors for developing peptic ulcer include *H. pylori* infection, alcohol and tobacco consumption, non-steroidal anti-inflammatory drugs (NSAIDs) use, and Zollinger–Ellison syndrome [5]. The main risk factors for both gastric and duodenal ulcers are *H. pylori* infection and NSAID use [6]. However, only a small proportion of people affected with *H. pylori* or using NSAIDs develop peptic ulcer disease, meaning that individual susceptibility is important in the beginning of mucosal damage. Functional polymorphisms in different cytokine genes are associated with peptic ulcers. For example, polymorphisms of interleukin 1 beta (*IL1B)* affect mucosal interleukin 1β production, causing *H. pylori*-associated gastroduodenal diseases [7].

On the other hand, the risk of complications of peptic ulcer is increased four times in NSAID users, and two times in aspirin users [8]. The concomitant use of NSAIDs or aspirin with anticoagulants, corticosteroids, and selective serotonin reuptake inhibitors increase the risk of upper gastrointestinal bleeding [9]. Although many people who use NSAIDs or aspirin have concurrent *H. pylori* infection, their interaction in the pathogenesis of peptic ulcer disease remains controversial. A meta-analysis of observational studies resulted in a conclusion that NSAIDs, aspirin use, and *H. pylori* infection increase the risk of peptic ulcer disease independently [10].

*H. pylori*-negative, NSAID-negative, and aspirin-negative peptic ulcer disease, which is classified as an idiopathic ulcer, can be diagnosed in about one-fifth of cases [11]. It is caused by the imbalance between factors that contribute to mucosal integrity and aggressive insults, but the pathogenic mechanisms behind the development of idiopathic peptic ulcer are still unknown [5]. A Danish study showed that psychological stress could increase the incidence of peptic ulcer [12]. Other etiologies include ischemia, drugs (steroids, chemotherapeutic agents) and radiotherapy, viruses, histamine, eosinophilic infiltration, gastric bypass surgery, and metabolic disturbances [13].

## 2. Pathogenesis of Peptic Ulcer

Almost half of the world’s population is colonized by *H. pylori*, which remains one of the most common causes of peptic ulcer disease [14]. The prevalence of *H. pylori* is higher in developing countries, especially in Africa, Central America, Central Asia, and Eastern Europe [15]. The organism is usually acquired in childhood in an environment of unsanitary conditions and crowding, mostly in countries with lower socioeconomic status. *H. pylori* causes epithelial cell degeneration and injury, which is usually more severe in the antrum, by the inflammatory response with neutrophils, lymphocytes, plasma cells, and macrophages.

The mechanism by which *H. pylori* induces the development of different types of lesions in the gastroduodenal mucosa is not fully explained. *H. pylori* infection can result in either hypochlorhydria or hyperchlorhydria, thus determining the type of peptic ulcer. The main mediators of *H. pylori* infection are cytokines that inhibit parietal cell secretion, but *H. pylori* can directly affect the H^+^/K^+^ ATPase α-subunit, activate calcitonin gene-related peptide (CGRP) sensory neurons linked to somatostatin, or inhibit the production of gastrin [16]. Although the formation of gastric ulcers is associated with hyposecretion, 10–15% of patients with *H. pylori* infection have increased gastric secretion caused by hypergastrinemia and reduced antral somatostatin content [17]. This leads to increased histamine secretion, and subsequently the increased secretion of acid or pepsin from parietal and gastric cells. Additionally, the eradication of *H. pylori* leads to a decrease in gastrin mRNA expression and an increase in somatostatin mRNA expression [18]. In the remaining majority of patients, gastric ulcers are associated with hypochlorhydria and mucosal atrophy.

The main mechanism of NSAID-associated damage of the gastroduodenal mucosa is the systemic inhibition of constitutively expressed cyclooxygenase-1 (COX-1), which is responsible for prostaglandin synthesis, and is associated with decreased mucosal blood flow, low mucus and bicarbonate secretion, and the inhibition of cell proliferation. NSAIDs inhibit the enzyme reversibly in a concentration-dependent manner. The co-administration of exogenous prostaglandins and cyclooxygenase-2 (COX-2)-selective NSAIDs use reduces mucosal damage and the risk of ulcers [19]. However, the different physicochemical properties of NSAIDs cause differences in their toxicity [20]. NSAIDs disrupt mucus phospholipids and lead to the uncoupling of mitochondrial oxidative phosphorylation, thus initiating mucosal damage. When exposed to acidic gastric juice (pH 2), NSAIDs become protonated and cross lipid membranes to enter epithelial cells (pH 7.4), where they ionize and release H^+^. In that form, NSAIDs cannot cross the lipid membrane, and are trapped in epithelial cells, leading to the uncoupling of oxidative phosphorylation, decreased mitochondrial energy production, increased cellular permeability, and reduced cellular integrity. Patients who have a history of peptic ulcers or hemorrhage, are over the age of 65, also use steroids or anticoagulants, and take high doses or combinations of NSAIDs are at the highest risk for acquiring NSAID-induced ulcers [1]

**Figure 1:**
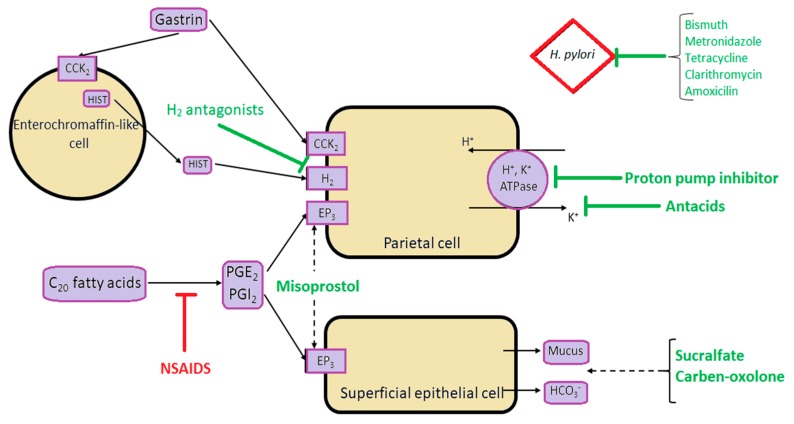
Schematic presentation of main pathophysiological mechanisms involved in the development of peptic ulcer disease, and the sites of action of the most commonly used pharmacological options in the treatment of peptic ulcer disease. CCK_2_ = Cholecystokinin Receptor; PGE_2_ = Prostaglandin E_2_; PGI_2_ = Prostaglandin I_2_; EP_3_ = Prostaglandin E receptor 3; HIST = Histamine.

Main pathophysiological mechanisms and the sites of action of antiulcer treatment are shown in the Figure 1.

## 3. Treatment

An overview of conventional antiulcer treatment options is summarized in Table 1 and Table 2.

### 3.1. Helicobacter pylori Eradication

Although successful *H. pylori* eradication alone is paramount for healing associated peptic ulcers and preventing relapses, the growing prevalence of antibiotic resistance made it a global challenge. The first effective therapy was introduced in the 1980s, and consisted of a combination of bismuth, tetracycline, and metronidazole that was given for two weeks [14]. The standard first-line therapy is a triple therapy consisting of a proton pump inhibitor (PPI) and two antibiotics, such as clarithromycin plus amoxicillin or metronidazole given for seven to 14 days [32]. However, with an increasing prevalence of antibiotic resistance, especially for clarithromycin, there has been a marked decline in the success of triple therapy over the last 10–15 years. *H. pylori* eradication should be based on antimicrobial susceptibility tests. As susceptibility testing is often not available in clinical practice, the choice of first-line therapies should be based on the local prevalence of antibiotic resistance, and clarithromycin-based regimens should be abandoned in areas where the local clarithromycin resistance rate is more than 15% [36]. The rate of eradication can be increased with the use of high-dose PPI and by extending the duration to 14 days [37].

The recommended standard first-line therapy is either a bismuth-containing quadruple therapy for 14 days (PPI, a bismuth salt, tetracycline, and metronidazole) or a 14-day concomitant therapy for patients intolerant of bismuth (PPI, clarithromycin, amoxicillin, and metronidazole); both regimens yield eradication rates higher than 90% [38].

Second-line therapy is prescribed if a first-line regimen fails, and should not include metronidazole or clarithromycin [39]. Levofloxacin triple therapy (PPI, amoxicillin, and levofloxacin) for 14 days seems to be an efficacious therapy, achieving eradication rates between 74–81% [33]. If a patient received first-line treatment with a clarithromycin-based regimen, a preferred treatment option is a bismuth quadruple therapy with eradication rates of 77–93%, or a high-dose dual-therapy regimen with amoxicillin and a PPI, as *H. pylori* rarely develops amoxicillin resistance [34]. Despite well-developed recommendations for choosing proper treatment regimens, 5–10% of patients have persistent infection. The most common reasons for the failure of two treatments are suboptimal compliance or the resistance of *H. pylori* to one or more antibiotics, in which case susceptibility testing is strongly recommended.

When at least three recommended options have been unsuccessful, one of the commonly recommended salvage regimens is rifabutin-based triple therapy (PPI, rifabutin, and amoxicillin) for 10 days, with eradication rates of 66–70% [35], but rifabutin’s adverse effects such as myelotoxicity and red secretions should be taken into account [40].

### 3.2. NSAID-Associated Ulcer Disease and the Use of PPIs

Many strategies are available for the prevention of NSAID and aspirin-associated gastroduodenal ulcers and their complications, such as the co-therapy of NSAIDs with a PPI, H₂ receptor antagonist, or misoprostol; the use of COX-2-selective NSAIDs; or their combination with a gastroprotective agent. PPIs are the most popular and effective prophylactic agents [41]. The mechanism of action is reducing the production of gastric acid through irreversible binding to the hydrogen/potassium ATPase enzyme on gastric parietal cells. The combination of COX-2-selective NSAIDs and a PPI offers the best protection against peptic ulcer complications [42]. Standard doses of H₂ receptor antagonists cannot reduce the risk of gastric ulcers [43]. Gastrointestinal upset and abortifacient actions limit the use of misoprostol for gastric protection, despite its effective prevention of peptic ulcer complications. Ulcers heal in more than 85% of cases with six to eight weeks of PPI therapy if the offending agent is discontinued. All of the gastric ulcers require repeat endoscopy to evaluate the success of healing. If ulcers fail to heal, drug compliance should be checked. For refractory ulcers, the doubling of PPI dose for another six to eight weeks is often recommended, although the evidence supporting this is weak. After the exclusion of false-negative *H. pylori* status, unusual causes of peptic ulcer should be explored, such as malignancies, infections, Crohn’s disease, vasculitis, upper abdominal radiotherapy, cocaine use, and Zollinger–Ellison syndrome.

PPIs are among the most commonly used and overprescribed medications in the world [44]. The side effects of the PPIs, such as a headache, diarrhea, constipation, and abdominal discomfort, are minor and easily managed. However, recent studies have suggested an association between PPI use and several serious adverse effects, which has been a source of major concern to patients and physicians. Some of the adverse effects of PPIs are related to their suppression of gastric acid secretion, allowing ingested microbial pathogens that would have been destroyed by gastric acid to colonize the upper gastrointestinal tract and cause infections. Reports are suggesting that the use of PPIs might increase the risk of enteric infections such as Salmonella and Campylobacter, community-acquired pneumonia [45], Clostridium difficile infections [46], and spontaneous bacterial peritonitis [47].

With gastric acid suppression, there is no stimulation of endocrine D cells to produce somatostatin, and thereby no inhibition of G cells for gastrin release, resulting in hypergastrinemia. Gastrin is a growth factor that can increase proliferation in Barrett metaplasia and the colon [48]. Nonetheless, PPI-induced hypergastrinemia in humans generally is mild, and rarely causes carcinoid tumors in human patients unless they have a genetic abnormality [49]. Furthermore, PPI usage might protect against cancer in Barrett’s esophagus, since PPIs heal the chronic esophageal inflammation of reflux esophagitis, which is a risk factor for the development of malignancy.

Gastric acid inhibition by PPIs also can affect the uptake of certain vitamins, minerals, and medications. There are reports of patients on PPIs developing vitamin B12 deficiency and iron deficiency anemia [50]. Additionally, PPIs might increase the risk for osteoporosis and bone fractures by interfering with the ionization and solubilization of the calcium salts that are required for their absorption [51]. The underlying mechanism for hypomagnesemia is still not clear. PPI-induced gastric acid suppression decreases ketoconazole absorption and facilitates the absorption of digoxin [52]. Furthermore, PPIs can affect the metabolism of other drugs metabolized by the cytochrome (CYP) P450 system; for instance, they can delay the clearance of warfarin, diazepam, and phenytoin. Considerable attention has been given to the potential of PPIs to reduce the antiplatelet action of clopidogrel, since both are metabolized by the CYP2C19 enzyme [53]. The clinical importance of the interaction remains disputed, but the Food and Drug Administration (FDA) has issued warnings to avoid using omeprazole or esomeprazole with clopidogrel.

There has been a dramatic increase in reports of miscellaneous, unanticipated adverse effects of PPIs over the past several years, such as myocardial infarction, stroke, acute and chronic kidney disease, and eosinophilic esophagitis. The increased frequency of cardiovascular events in patients on clopidogrel who also use PPIs can be the result of the drugs competing for metabolism by CYP2C19, although there is a possibility that PPIs might have cardiovascular effects that are independent of their effects on clopidogrel activation, perhaps by the decreased production of nitric oxide and altered vascular homeostasis [54]. It has been proposed that PPIs might contribute to the development of eosinophilic esophagitis through their effects on peptic digestion [55]. The suppression of acid production raises gastric pH and inactivates pepsin, inhibiting peptide ingestion and degradation, and causing allergic reactions in the small intestine.

### 3.3. Potassium-Competitive Acid Blockers

Since up to 13% of patients treated with lansoprazole still experience ulcer recurrence, the search for alternative treatment is ongoing. Vonoprazan is a potassium-competitive acid blocker that inhibits H^+^, K^+^-ATPase in gastric parietal cells at the final stage of the acid secretory pathway [25]. The difference in the mechanism of action between vonoprazan and PPIs is that vonoprazan inhibits the enzyme in a K^+^-competitive and reversible manner, and does not require an acidic environment for activation. Additionally, vonoprazan shows a rapid onset of action and prolonged control of intragastric acidity [26]. Vonoprazan at doses of 10 mg and 20 mg was non-inferior to lansoprazole for the prevention of peptic ulcer recurrence in Japanese patients during NSAID therapy [25], or those who required aspirin therapy for cardiovascular or cerebrovascular protection [27], with good tolerance, a similar safety profile, and no new safety issues. Also, five weeks of treatment with vonoprazan significantly reduced post-endoscopic submucosal dissection bleeding, compared to eight weeks of treatment with PPIs [28]. Similarly, it was shown to be superior to esomeprazole [29] and rabeprazole [26] for scarring artificial ulcers, which could help make an endoscopic submucosal dissection a safer treatment.

### 3.4. Future Research Questions

Along with the global decline of peptic ulcer disease and in the prevalence of *H. pylori*, there is a rising problem of growing antimicrobial resistance, which reduces the efficiency of eradication therapy, and the overuse of PPIs, resulting in unexpected new side effects [56]. Also, the occurrence of idiopathic ulcers associated with high mortality is increasing [57], and there is a need for defining the optimum management of the idiopathic disease. There is still an open question of how *H. pylori* infection and NSAID or aspirin interact, leaving the best strategy to manage patients with both risks unresolved. The pathogenesis of *H. pylori*-related gastric lesions is still not fully understood. Its development is led by a combination of *H. pylori* virulent factors and the host immune response; however, the precise combination of *H. pylori* factors and the host genetic profile are yet to be fully enlightened. Why some patients are more susceptible than others to the gastric toxicity of NSAIDs and aspirin, and which genetic polymorphisms are associated with *H. pylori-*induced peptic ulcer also remain unclear.

In the absence of any possible breakthrough antimicrobial agent for *H. pylori*, antibiotic resistance continues to be a major challenge, and new therapies are in fact old therapies. *H. pylori* urease has been at the center of attention for the development of antiulcer treatment. Several potent in vitro inhibitors have been found, but with poor specificity. They usually don’t make it to the clinical setting due to the high dosage required, increased cost of treatment, and increased risk for bleeding. Recent advances in the molecular description of *H. pylori* pathogenesis resulted in promising candidates related to the pathogen’s persistence in the host, such as adherence. Some antivirulence agents can selectively target the pathogen’s adherence, but a high binding affinity and genetic diversity in the receptor-binding site of *H. pylori* complicate the finding of potent inhibitors [58].

The genetic diversity of the virulence proteome in *H. pylori* direct future antivirulence developments toward its more conserved assembly and secretion pathways, leaving the open question of how these inhibitors can contribute to *H. pylori* treatment.

Gastrointestinal bleeding as the complication of peptic ulcer disease remains life-threatening, and comorbidities are now the primary cause of death in these patients. There is an urgent need for prospective data and randomized controlled trials to define the best patient care strategy. In the meantime, appropriate diagnostics, adherence to current guidelines, and the avoidance of inferior *H. pylori* treatment regimens will be necessary to maintain successful treatment of peptic ulcer.

## 4. Alternative Therapy for Peptic Ulcer

The usage of medicinal plants in healing numerous diseases is as old as human beings, and well-known as phytotherapy. Moreover, in the past few years, there has been a rising interest in alternative therapies and the usage of herbal products, in particular, those produced from medicinal plants [59,60]. Also, due to appearance of various side effects by usage of conventional drugs for numerous diseases, medicinal plants are considered the major reservoir of potentially new drugs. Plant extracts and their crude are the most significant sources of new drugs, and have been shown to cause promising results in the treatment of gastric ulcer as well [61]. It is known that numerous pharmaceutical agents such as proton pump inhibitors, anticholinergics, antacids, antimicrobial agents, H2-receptor antagonists, sucralfate, and bismuth are not fully effective, and produce numerous adverse effects such as impotence, arrhythmia, hematopoietic alterations, hypersensitivity, and gynecomastia [62,63]. Due to that, investigations of the new pharmacologically active agents through the screening of different plant extracts led to the discovery of effective and safe drugs with gastroprotective activity. Especially, plants with antioxidant capability as the main mechanism are used as the herbal reservoir for the treatment of ulcer disease [63].

Medicinal plants have achieved their therapeutic properties from their capability to produce renewable and various secondary metabolites, which are known as phytochemical constituents. Hence, numerous plants have used these phytochemicals as a protection mechanism against pathogens [64].

On the other hand, the appearance of resistant pathogens has had a significant influence on the pharmaceutical companies to change their strategy in the development of conventional antibiotics and design new antimicrobial drugs derived from medicinal plants [65]. Nevertheless, the synthetic antibiotics are still dominant as antimicrobial drugs.

As a matter of fact, incidences of infectious diseases have enlarged within the last three decades, involving infections with different properties as well as new infections, and it has been shown that around 60% of them are of zoonotic origin (spread among human and animals). *H. pylori* is one of the major representatives in that group, and may cause chronic gastritis, peptic ulcer disease, and stomach cancer [66]. Therefore, one of the aims in this review was to highlight some medicinal plants that demonstrated significant antibacterial and antioxidant activity against *H. pylori* and peptic ulcer disease. However, some of plants lose their efficiency against *H. pylori* consequent to the emergence of resistant strains. Consequently, the isolation of various constituents from the most active plant extracts is encouraged [67].

It is important to emphasize that herbal products may contain numerous bioactive constituents with dangerous, but also beneficial effects. Therefore, the higher education of doctors and patients about herbal therapy is necessary, as well as legislation to control the quality of herbal products, especially for further randomized investigations to determine the effectiveness and safety of many products in digestive and other disorders [68].

Finally, the Ayurvedic knowledge and modern medicine could generate preferable antiulcer drugs derived from medicinal plants with less side effects [69].

Numerous medicinal plants with significant antibacterial activity against *H. pylori* and benefits for gastric ulcer disease are shown in Table 3.

### 4.1. The Effect on H. pylori Eradication

Several factors influence the conventional therapy failure. These include: the poor bioavailability of antibiotics, as the gastric mucus layer plays a barrier to antibiotic delivery, and therefore the drugs are unable to obtain the underlying gastric epithelium [70]; the stomach containing a pH from acidic to neutral, and only a few antibiotics are active in a wide pH range [79]; bacterial antagonism to antibiotics, where co-infection with multiple strains is quite an important feature [80]; deficiency of patient permissiveness to the therapy; patients lifestyle, and diet [46].

Numerous studies have been reported about various medicinal plants and their anti-*H. pylori* activity. In recent years, it has been shown that the suppression of enzymatic (dihydrofolate reductase, DNA gyrase, myeloperoxidase N-acetyltransferase, and urease) and adhesive activities, the high redox potential, and hydrophilic/hydrophobic natures of constituents have a significant role in anti-*H. pylori* action mechanisms. *H. pylori*-stimulated gastric inflammation may lead to superficial gastritis and atrophic gastritis, but also to gastric cancer. It is established that different natural products have anti-inflammation activity, and the fundamental mechanisms involve the inhibition of nuclear factor-κB and mitogen-activated protein kinase pathway activation and the suppression of oxidative stress.

Since the role of *H. pylori* infection regarding carcinogenesis is to ascend carcinogenesis instead to play a key role as a direct carcinogen, its eradication alone cannot inhibit *H. pylori*-related gastric cancers [81].

Medical plants such as Allium sativum, Zingiber officinalis, Korean red ginseng, and Cistus laurifolius are known to suppress the colonization of *H. pylori*, reduce gastric inflammation by chemokine release, inhibit cytokine, and suppress precancerous changes by suppressing nuclear factor-kappa B DNA binding, which suppresses mutagenesis and produces abundant levels of apoptosis. Further unresolved issues will have to be cleared out before phytoceuticals are accepted as a standard therapy for *H. pylori* infection [82].

### 4.2. Korean Red Ginseng

*Korean red ginseng* extract plays a significant role in inhibiting *H. pylori*-induced 5-LOX activity, such as inactivating c-jun, repressing NF-κB-DNA binding, inhibiting *H. pylori*-induced 5(S)-hydroxyeicosatetraenoic acid biosynthesis, and preventing pro-inflammatory interleukin (IL)-8 or 5-LOX mRNA. Consequently, these mechanisms decrease gastric carcinogenesis. 

Moreover, *Korean red ginseng* has been shown to be beneficial in suppressing 5-lipoxygenase (5-LOX) mRNA and enzyme activities, and consequently the decreased synthesis of 5-hydroxy-eicosatetraenoic acid. Similarly, green tea extract may prevent the activation of multiple transcription factors and their target genes, involving COX-2 and inducible nitric oxide synthase (iNOS) mitogen-activated protein kinase activation, as well as the lipopolysaccharide of *H. pylori*-activated TLR-4. Due to that, these blockades increase the pro-inflammatory factors that induce gastric mucosal lesions [83,84]. Kim et al. reported on the protective effect of *Korean red ginseng* against *H. pylori*-induced cytotoxicity in vitro [83]. Meanwhile, in a previous clinical study, a supplementary administration of *Korean red ginseng* increased the eradication rates of *H. pylori*, reduced gastric inflammation, and decreased oxidative DNA damage and apoptosis [84].

### 4.3. Allium Sativum

Throughout history, the health benefits of garlic have been well documented, and the main use of *Allium sativum* was for its medicinal properties. The organosulfur components of *Allium sativum*, including S-allyl-L-cysteine (SAC) sulfoxides and δ-glutamyl S-allyl-L-cysteine, are known as main compounds of its bioactivity. Raw *Allium sativum* is easy to convert in bioinactive form. Accordingly, numerous types of its extract with different compositions of bioactive components have been developed, and their efficacy has been observed and evaluated in numerous studies [85]. The major role of *Allium sativum* extract has been observed in antioxidant effect by scavenging reactive oxygen species (ROS), inhibiting lipoprotein oxidation and lowering the serum glucose induction of antioxidant enzymes. Also, it showed a suppressive effect of *H. pylori*-induced gastric inflammation in vivo [86], and an anti-tumorigenic effect by promoting apoptosis and the induction of cell cycle arrest [87]. Allicin and allyl-methyl plus methyl-allyl thiosulfinate from acetonic *Allium sativum* extracts have restricted the growth of *H. pylori* in the in vitro investigations [88].

### 4.4. Cistus Laurifolius

Flavonoids are one of the most important components of the human diet with a key role in organisms and significant responsibility for numerous biological activities, in particular, antioxidant. Due to their limited availability and high cost, a rapid synthesis of polyoxygenated flavones, starting from accessible and inexpensive flavanones, has been developed. By methoxylation and bromination protocol 3′-demethoxysudachitin, a restricted flavone with antimicrobial activity against *H. pylori* has been designed. Numerous investigations on flavoinoids were done with an extract of *Cistus laurifolius*. It has been demonstrated when testing for antimicrobial activity against *H. pylori* that 3’-demethoxysudachitin and sudachitin were the most active compounds. A similar investigation showed that the chloroform extract of *Cistus laurifolius* has tremendous anti-*H. pylori* activity. Accordingly to these investigations, isolated flavonoids can be used as an additive component for the standard treatment of *H. pylori* infection [82,89].

Li HQ et al. observed diverse levels of anti-*H. pylori* activities in numerous isoflavones [90]. The experiment evaluated a few series of metronidazole-flavonoid extracts that have been used for antimicrobial activity against *H. pylori* [90]. It has been demonstrated that only one compound could remarkably achieve the enhancement in IL-8 levels in the gastric cancer cells induced with a *H. pylori* water extract. On the other hand, Nakagawa et al.’s experiments revealed that new flavonoid compounds 6, 7, and (2S)-4′,7-dihydroxy-8-methylflavan were discovered to be most efficacious compounds against *H. pylori* [91].

Similarly, Ustun et al. discovered that the chloroform extract of *Cistus laurifolius* holds a significant anti-*H. pylori* effect [42]. Accordingly, isolated flavonoids can be used as an alternative or supplement compound to the current treatment of *H. pylori* infection [76].

### 4.5. Zingiber Officinalis and Zingiber Zerumbet

*Zingiber officinalis* is known as ginger, which is consumed as a flavoring agent. The plant extract showed antitumor effects on colon cancer cells by inhibiting its growth, increasing DNA synthesis, and producing apoptosis [92]. Moreover, the main pungent phenolic compound of *Zingiber officinalis* is 6-gingerol, which has numerous pharmacological activities. *Zingiber officinalis* extracts containing gingerols have key role in prostaglandin E2 (PGE2) inhibition [73]. On the other side, the active phenolic compounds such as gingerol and zingerone have a significant influence in inhibiting parietal cell H^+^, K^+^-ATPase. Due to that, the activity of gingerol and zingerone plays a very important role in proton pump inhibition and the reduction of gastric acid secretion. Also, it shows a protective effect against *H. pylori*-induced ulcers [74].

Jiang et al. demonstrated the therapeutic effect of *Zingiber officinalis* as a natural antioxidant against gastric ulcers [93]. They reported free *Zingiber officinalis* extracts limitations such as slight solubility in gastric juices, which will reduce further as it passes to higher pH regions of duodenum or ileum in rats; numerous medicaments show a restricted transit time of less than two to four hours in the stomach; whichever part is solubilized will be instantly absorbed, because *Zingiber officinalis* extract indicates fast absorption, consequently, local therapeutic effect cannot be elicited adequately [93].

In addition, Sidahmed et al. showed that zerumbone from *Zingiber zerumbet* has a major role in gastroprotection activity against ethanol-induced gastric ulcer model in rats. They demonstrated that pretreatment with zerumbone or omeprazole in rats significantly reduced ulcer area formation compared to the ulcer control group. Moreover, pretreatment with omeprazole at 20 mg/kg body weight (b.w.) (*p* < 0.05) obstructed formation of ulcer by 76.77%, while pretreatment of zerumbone at five and 10 mg/kg b.w. obstructed ulcer formation by 75.59% and 88.75%, respectively. On the other hand, zerumbone and its gastroprotective mechanisms were not tested against other ulcer model; hence, other mechanisms may be implicated and their influence needs to be investigated and elucidated [94].

### 4.6. Camellia Sinensis (Green Tea Polyphenols)

Nowadays, *Camellia sinensis* is one of the most commonly used beverages. The chemopreventive effects of *Camellia sinensis* depend on its activity as an antioxidant, but also on its molecular regulatory functions on cellular growth, development, and apoptosis; and a selective improvement in the function of the intestinal bacterial flora. Between the numerous constituents of green tea, polyphenols and epigallocatechin gallate (EGCG) suppress tumor necrosis factor-alpha (TNF-α) gene expression [95]. On the other hand, the urease of *H. pylori* is crucial for its colonization, and investigations concentrated on *Camellia sinensis* extract demonstrated the inhibitory activity of this enzyme. That results in the inhibition of bacterial colonization [96]. Numerous similar studies demonstrated the inhibitory effect of *Camellia sinensis* extract by increasing cell vacuolation by vacuolating cytotoxin A (vacA) and urea conduction in *H. pylori* infection. Consequently, it could pursue anti-*H. pylori* activity in vivo [97].

In 2008, Rao et al. reported on the gastroprotective activity of 50% ethanolic extract of *Ficus glomerata fruit* (FGE) in gastric ulcer models in rats [98]. FGE was administered per mouth (50, 100, and 200 mg/kg body weight), twice daily for five days for prevention from ethanol (EtOH), pylorus ligation (PL), and cold restraint stress (CRS), which induced ulcer formation. It demonstrated a dose-dependent suppression of ulcer, and it had a significant role in preventing the oxidative damage of gastric mucosa by preventing lipid peroxidation and significantly reducing in H^+^/K^+^-ATPase and superoxide dismutase. Their results showed that *F. glomerate* has an important gastroprotective effect that might be consequent to the gastric defense factors [98].

### 4.7. Curcuma Longa and Artemisia Asiatica

Medicinal plants with antioxidant and anti-inflammatory activity have had a demonstrated effect on gastroesophageal reflux disease (GERD). The medicinal plants and herbal preparations with antioxidant and anti-inflammatory mechanisms include *Curcuma longa*, *Panax quinquefolium, Artemisia asiatica*, and *Lonicera japonica*. Moreover, other mechanisms include: the down-regulation of the genes encoding proteins that have key role in acute inflammation, including 1 intercellular adhesion molecule-1 (ICAM-1) and cytokine-induced neutrophil chemoattractant-2-beta (CINC-2-2 beta) (*Panax quinquefolium*); ameliorating the function and gastric mucus (*Morus alba, Curcuma longa*); reducing gastric acid, such as for instance *Curcuma longa*, *Morus alba*, and acidinol syrup, increasing tonic contractions of the lower esophageal sphincter (LES) (*Salvia miltiorrhiza*, STW 5), and preventing the pro-inflammatory cytokines IL-1 b and TNF-a (STW 5) [99].

It is important to mention investigation on rats where pretreatment with compounds of *Artemisia asiatica* (DA-9601) reduced the overall density of the esophageal wall and volume of ulceration beyond the ranitidine group [100].

Mahattanadul showed in his study on rats that the rhizome of *Curcuma longa* plays a protective role in the formation of acute acid reflux esophagitis (RE), but it was not effective in the prevention of chronic acid RE [101]. However, its combination with dimethyl sulfoxide as an antioxidant compound reduced the severity of the esophagitis ulcer index to around that of lansoprazole. In contrast, lansoprazole inclined to elevate the severity of all histopathological changes above the control and curcumin-treated groups. Hence, it seemed that the antioxidant and anti-inflammatory activity of curcumin plays a major role in its beneficial effects on GERD [101].

Herbal medicine can be a mighty weapon for suppressing or modulating the disease-associated footprints of *H. pylori* infection and eradication. Finally, those plant products have shown strong potential as pharmaceutical candidates in gastric disease prevention [68].

## 5. Herb–Drug Interactions

Together with increasing use of herbal supplements worldwide, the number of adverse events and drug interactions is rising. Interactions between an herbal supplement and a drug can manifest as a pharmacokinetic or pharmacodynamic interaction. Pharmacokinetic interaction is a result of using the same mechanism of absorption, distribution, metabolism, or excretion between an herbal supplement and a co-administered drug, leading to the change of the drug’s concentration in the blood and pharmacologic action. Pharmacodynamic interactions involve a direct effect on the mechanism of action of a co-administered drug without changing the drug’s concentration, only by antagonizing or exacerbating the drug’s clinical effects [77].

*Allium sativum* extract decreases concentrations of drugs transported by P-gp, such as digoxin, doxorubicin, rosuvastatin, and verapamil [102]. The most studied *Allium sativum* interactions is the one with warfarin, although this has not yet been confirmed by controlled clinical trials. Also, it inhibits platelet aggregation, so it should be used with caution in patients with clotting disorders or those with anticoagulant therapy [103]. *Zingiber officinalis* prolongs bleeding time by the inhibition of thromboxane synthetase, but this has not been confirmed in a clinical trial [104]. *Ginkgo biloba* could increase bleeding risk, especially in combination with anticoagulant drugs, due to the inhibition of platelet aggregation. Flavonoids in *Ginkgo biloba* have antiplatelet activity, but do not affect blood coagulation or platelet function in humans [103]. In combination with NSAIDs, it can cause severe bleeding [105].

*Panax ginseng* induces cytochrome P450 3A4 (CYP3A4), which decreases the effectiveness of calcium channel blockers, certain antihypertensive and statin medications, and some antidepressants [106]. *Panax ginseng* has hypoglycemic activity in patients with diabetes, and may cause headache, trembling, and manic behavior in patients treated with phenelzine [107].

Green tea extract has been shown to increase simvastatin concentrations [108], or inhibit the drug transporters organic anion transporting protein 1a1 (OATP1A1) and anion transporting protein 1a12 (OATP1A2), which are responsible for the transport of fluoroquinolones, beta blockers, and imatinib [77].

Of the conventional antiulcer treatment, it is important to emphasize the many drug interactions of cimetidine [109]. Studies have reported clinically important interactions with warfarin, phenytoin, diazepam, chlormethiazole, propranolol, lidocaine, and a number of other drugs [110]. Also, cimetidine can increase the level or effect of green tea due to CYP1A2 inhibition, which consequently inhibits the hepatic oxidative metabolism of caffeine [111].

## 6. Conclusions

The combination of herbal products and standard anti-gastric ulcer drugs might present a synergistic effect against *H. pylori* and gastric ulcer disease and improve the outcome for patients with gastric ulcer. With only a few human studies, it is suggested to conduct further clinical studies with larger sample sizes on the efficacy and safety of medicinal plants with antiulcer activity. Also, it would be beneficial to design studies to investigate and further elucidate the mechanisms of action of medicinal plants used for the treatment or prevention of peptic ulcer.

Finally, herbal products used for medicinal purposes require licensing in order to ameliorate their safety and quality, and ensure that randomized controlled investigations validate demands of its possible efficacy. With increased reports of herb–drug interactions, there is still a problem of deficient research in this field, with no measures taken to address this problem. Hence, pharmacists and doctors should be aware especially of the risks associated with the usage of herbal preparations, whether on their own or in combination with other herbal or standard conventional therapy.

## Figures and Tables

**Table 1:** Mechanisms of action and adverse effects of the most commonly used antiulcer treatment options.

Medicine		Mechanism of Action	Adverse Effects	References
**Proton Pump Inhibitors (PPIs)**	Omeprazole	Inhibition of the gastric H^+^/K^+^-ATPase (proton pump) enzyme system	Headache Abdominal pain Diarrhea Nausea Vomiting Constipation Flatulence Vitamin B12 deficiency Osteoporosis	[21,22]
	Lansoprazole			
	Rabeprazole			
	Esomeprazole			
	Pantoprazole			
**H2 Receptor Blockers**	Cimetidine	Blocking the action of histamine at the histamine H2 receptors of parietal cells	Headache Anxiety Depression Dizziness Cardiovascular events Thrombocytopenia	[23]
	Famotidine			
	Nizatidine			
	Ranitidine			
**Antacids**	Aluminum hydroxide	Increases gastric pH to greater than four, and inhibits the proteolytic activity of pepsin	Frequency not defined: Nausea Vomiting Hypophosphatemia Chalky taste Constipation Abdominal cramping Diarrhea Electrolyte imbalance	[24]
	Magnesium hydroxide	Causes osmotic retention of fluid		
**Potassium-Competitive Acid Blocker**	Vonoprazan	Inhibits H^+^, K^+^-ATPase in gastric parietal cells at the final stage of the acid secretory pathway	Nasopharyngitis Fall Contusion Diarrhea Upper respiratory tract inflammation Eczema Constipation Back pain	[25,26,27,28,29]
**Cytoprotective Agents**	Misoprostol	Stimulate mucus production and enhance blood flow throughout the lining of the gastrointestinal tract	Diarrhea Abdominal pain Headache Constipation	[30,31]
	Sucralfate			

**Table 2:** Types and efficiency of *Helicobacter pylori (H. pylori)* eradication treatment options.

Type	Duration	Efficiency	References
**First line**			
*Standard triple therapy*:			
PPI + two antibiotics (clarithromycin + metronidazile or amoxicilin)	7–14 days	70–85%	[32]
**Second line**			
*Bismuth-containing quadruple therapy:*			
PPI + bismuth salt + tetracycline + metronidazole	14 days	77–93%	[33,34]
*Non-bismuth based concomitant therapy:*			
PPI + clarithromycin + amoxicillin + metronidazole	14 days	75–90%	
*Levofloxacin triple therapy:*			
PPI + amoxicillin + levofloxacin	14 days	74–81%	
**Salvage regimens**			
*Rifabutin-based triple therapy:*			
PPI + rifabutin + amoxicillin	10 days	66–70%	[35]

PPI: proton pump inhibitors.

**Table 3:** Overview of herbal antiulcer treatment and *H. pylori* eradication.

Medicinal Plant	Possible Mechanisms	Effect	Adverse Effects	References
*Korean red ginseng*	Inhibition of *H. pylori*-induced 5-lipoxygenase (5-LOX) activity; preventing pro-inflammatory interleukin (IL)-8 or 5-LOX mRNA	Anti-inflammatory effect; increase eradication rates of *H. pylori*; reduction of gastric inflammation and oxidative DNA damage	Interaction with conventional drugs	[69,70]
*Allium sativum*	Inhibition of lipoprotein oxidation and lower serum glucose induction of antioxidant enzymes; mechanisms need to be more investigated	Antioxidant; suppressive effect of *H. pylori*-induced gastric inflammation in vivo and in vitro	Interaction with conventional drugs	[71]
*Curcuma loga*	Inhibition of *H. pylori*-induced 5-LOX activity	Anti-inflammatory; antioxidant	Not determined	[72]
*Zingiber officinalis*	Inhibition of PGE2 and parietal cell H^+^, K^+^-ATPase	Anti-inflammatory effect; antioxidant	Nausea and vomiting in pregnant women; restless, heartburn; interaction with conventional drugs (anticoagulants, analgesics)	[73,74,75]
*Zingiber zerumbet*	Gastroprotective mechanism of zerumbone (significant increased in the endogenous antioxidant GSH, reduction of lipid peroxidation level); other mechanism need to be investigated	Antioxidant, antiproliferative, anti-inflammatory, antisecretory effect; reduction of ulcer area formation	Nausea and vomiting in pregnant women; restless, heartburn; interaction with conventional drugs (anticoagulants, analgesics)	[75,76]
*Camellia sinensis* (Green tea polyphenols)	Suppression of tumor necrosis factor-alpha (TNF-α) gene expression; inhibition of urease	Antioxidant; improvement in the function of intestinal bacterial flora	Interaction with conventional drugs; dizziness, diarrhea, headaches, insomnia, heartbeat, may cause deficiency of iron	[77,78]

## Abbreviations

IL1B	interleukin 1 beta
COX-1	cyclooxygenase 1
COX-2	cyclooxygenase 2
CYP	cytochrome
FDA	Food and drug administration
*H. pylori*	*Helicobacter pylori*
CCK2	cholecystokinin receptor
PGE2	prostaglandin E2
PGI2	prostaglandin I2
EP3	prostaglandin E receptor 3
HIST	histamine
H2 receptor agonists	histamine-2 receptor agonists
NSAIDs	non-steroidal anti-inflammatory drugs
PPIs	proton pump inhibtors
5-LOX	5-lypoxigenase
iNOS	inducible nitric oxide synthase
SAC	S-allyl-L-cysteine
EGCG	epigallocatechin gallate
vacA	vacuolating cytotoxin A
CRS	cold restraint stress
PL	pylorus ligation
GERD	gastroesophageal reflux disease
LES	lower esophageal sphincter
IL	interleukin
ROS	reactive oxygen species
TNF-α	tumor necrosis factor-alpha
ICAM 1	intercellular adhesion molecule-1
CINC 2-beta	cytokine-induced neutrophil chemoattractant-2-beta
OATP1A1	organic anion transporting protein 1a1
OATP1A2	anion transporting protein 1a2
STW 5	a complex herbal combination preparation composed of 9 different herbal extracts
CYP3A4	cytochrome P450 3A4
